# Reverse wedge effect following intramedullary nail fixation of trochanteric fracture, what does it imply?

**DOI:** 10.1186/s12891-021-04388-1

**Published:** 2021-05-29

**Authors:** Wei Hao, Long Fang, Shuangshuang Yin, Yongjie Lin, Bingchen Wang

**Affiliations:** 1 Department of Joint and Sports Medicine, Shandong Provincial Third Hospital affiliated with Shandong University, Jinan, China; 2 Department of Spine Surgery, Shandong Provincial Third Hospital affiliated with Shandong University, Jinan, China; 3 Department of Nursing, Shandong Provincial Third Hospital affiliated with Shandong University, Jinan, China

**Keywords:** Reverse wedge effect, Trochanteric fracture, Basicervical, Intraoperative, Mechanism

## Abstract

Lag screw cut-out is the most common cause of fixation failure of trochanteric fractures. Intraoperative assessment of fracture reduction and fixation quality is vital to avoid fracture reduction and achieve good functional outcomes. In a recent study, Zhang et al. reported the occurrence of a reverse wedge effect after intraoperative nail insertion based on a new computed tomography(CT)-guided fracture classification system, which specifically happened to the basicervical facture type and resulted in valgus deformity with gapping at the medial inferior fracture line. Impingement between the reamer/nail and superolateral cortex of the femoral neck has been regarded as the main cause. Based on these findings, together with an extensive literature review, the practicality of the new fracture classification system, the definition of basicervical trochanteric fracture, and the mechanisms underlying the reverse wedge effect have been deeply discussed. More studies should be carried out in the future to analyse pre- and intraoperative related factors that could affect the intraoperative fragment migration effects and determine highly specific measures to address them.

## Background

Currently, few studies have concentrated on the intraoperative assessment of fracture reduction and fixation quality during trochanteric fracture operations. However, it is critical to achieve a good prognosis and avoid complications. Zhang et al. recently published a study concerning the reverse wedge effect after intraoperative nail insertion based on a new CT-guided fracture classification system. This new intraoperative complication has been indicated to be exclusively related to the basicervical fracture type. Impingement between the reamer/nail and superolateral cortex of the femoral neck has been regarded as the main cause. Here, a more extensive literature review regarding the widely used Arbeitsgemeinschaftfür Osteosynthesefragen/Orthopaedic Trauma Association(AO/OTA) fracture classification system, wedge/reverse wedge effect, stable/unstable trochanteric fracture, basicervical intertrochanteric fracture, and bony structure of the proximal femur is presented. The newly issued CT-based fracture classification system, the reverse wedge effect with the underlying mechanism, has been further discussed.

## Main text

With the ageing population increasing worldwide, osteoporotic hip fractures have continued to rise accordingly, among which approximately half cases are trochanteric fractures. Surgery with internal fixation (sliding hip screw or cephalomedullary devices) at an early time (within 48 h after injury) has been associated with better outcomes [1]. However, analyses concerning postoperative fixation failure indicate that lag screw cut-out is the most common cause [2]. Cut-out of the screw from the femoral head is defined as “the collapse of the neck-shaft angle into varus, leading to extrusion, or so-called cutout, of the screw from the femoral head” [3]. Theoretically, a vertical force would exist and pass downward from the centre of the femoral head, which tends to move the affected hip into varus when the patient stands postoperatively. Clinically, fixation in a more varus reduction has been proven to tend to have a higher lag screw cut-out rate [2, 4]. Therefore, intraoperative fracture reduction and fixation quality are critical to achieve favourable neck-shaft angle and avoid cox vara occurrence.

### Proposal of reverse wedge effect conception in addition to the wedge effect

The concept of the “wedge effect” was first proposed by Doctor O’Malley in 2015 [5] and is termed “include both femoral shaft lateralization and varus malalignment of the neck after nail insertion”. The results demonstrated up to 7 mm lateralization of the femoral shaft and 4° additional varus postoperation compared to the unaffected contralateral side, with no relationship to specific fracture types. This phenomenon can be taken as a type of reduction loss after nail insertion into the proximal femoral shaft. Several ways have been implemented to try to solve this problem, including medializing the entry point at the tip of the greater trochanter, intraoperative overdistraction, provisional fixation with Kirschner wires or pins, etc. [6]. However, those procedures have not worked as well as expected, especially for most unstable fractures, which could only achieve an ‘‘acceptable’’ reduction grade based on Baumgaertner reduction quality criteria [7].

Contrary to the conception of the wedge effect, Zhang et al. proposed the “reverse wedge effect” during intraoperative fracture reduction and fixation in a recent issue of the journal [8]. The reverse wedge effect is described as “the reaming/intramedullary nail(IN) insertion generated internal rotation of the cephalocervical fragment and an inferiorly oriented gap at the primary fracture line (basicervical region)”. They carried out a retrospective study concerning analyses of intraoperative fluoroscopy for trochanteric fractures. In total, 414 patients with unilateral fractures were enrolled. The proximal femur was divided into four parts: cephalocervical fragment, femoral shaft, posterolateral fragment from the greater trochanter and lesser trochanter. Based on CT examination, four fracture subtypes were classified accordingly. The results demonstrated that 33 cases with intraoperative reverse wedge effects belonged to the subtype of basicervical trochanteric fracture variants. Nevertheless, 12 cases among this specific fracture subtype did not present similar intraoperative findings, which accounted for up to 27 % of the total cases. Finally, impingement between the reamer/nail and superolateral edge of the cephalocervical fragment, with a compromised great trochanter, has been considered to be the underlying mechanism. However, several findings of this valuable study deserve further discussion and need to be viewed in the context of published literature in this area.

### Roles of lateral wall thickness

Based on the AO/OTA classification 2018 version [9], lateral wall thickness is clearly defined as “the distance in millimetres (mm) from a reference point 3 cm below the innominate tubercle of the greater trochanter angled 135° upward to the fracture line (midline between the two cortices) on the anteroposterior X-ray”. Hsu et al. proved that lateral wall thickness is a reliable predictor to evaluate the risk of secondary lateral wall fracture postoperation after dynamic hip screw (DHS) fixation [10]. They analysed 208 cases (AO/OTA A1, A2) with DHS fixation and found that when the lateral wall thickness was 20.5 mm, the highest sensitivity and specificity was achieved to estimate a threshold value that could predict lateral wall fracture based on the receiver operating characteristic (ROC) curve. In fractures of AO/OTA A1 with lateral wall thickness > 20.5 mm, DHS alone can be used because the relatively intact lateral wall can provide enough buttress effect on the outer side to prevent the fracture from excessive collapse. However, for fracture type AO/OTA A2 (lateral wall thickness < 20.5 mm), in which the posteromedial cortex at the fracture side loses continuity, the lateral wall bears more stress, and then secondary fracture ensues postoperation at a higher risk with DHS alone. Additionally, secondary fracture of the lateral wall will occur intraoperatively when the large-diameter hole for the sliding hip screw is drilled into it [11]. This leads to the use of intramedullary nails for fracture types with more vulnerable lateral walls [1]. Therefore, lateral wall thickness measurement based on plain X-ray has been justified to be simple, reliable, easy to reach and applied for not only classifying fracture type but also determining the choice of internal fixation device for various fracture types [12–15]. However, from the authors’ point of view, lateral wall thickness measurement is “unreliable and generated confusion in differentiating AO 31-A1 from A2” in AO/OTA classification 2018 version. A new CT-based fracture classification system has then been proposed, which mainly differs in what specified range around the greater trochanter fragment will be involved: small, moderate or moderate with lesser trochanter as a whole piece, greater trochanter with separated lesser trochanter fragment. Although this new CT-based fracture classification system is concrete and detailed, its roles as a possible predictor or instructor for pre-, intra- or postoperative fracture management remain lacking. Further prospective clinical studies should be performed to interpret its advantage over the AO/OTA classification system.

### Definition of unstable fractures includes basicervical trochanteric type

The definition of unstable fractures varies but should include the following aspects: lesser trochanter fragment, reverse fracture line or intertrochanteric comminution associated with a large posteromedial component, broken greater trochanter and lateral cortex breach [16]. Fractures with broken greater trochanter and/or lateral cortex breach can be easily defined as specific fracture subtypes and treated with adequate internal fixator according to AO/OTA classification 2018 version by measuring lateral wall thickness on plain X ray [9]. For the rest, the underlying mechanism leading to unstable fracture is the breakage of posteromedial cortex support, where the calcar femorale is located. The calcar is a vertical plate of dense cancellous bone that lies deep to the lesser trochanter but posterior to the neutral axis of the femoral neck. It is involved in weight bearing and forces transmission through the femoral neck [17]. Posteromedial cortex support has been accepted as an important factor for the stability of intertrochanteric fractures [18]. Therefore, the fractured lesser trochanter can be taken as a sign that is equivalent to the absence of medial calcar, i.e., unstable fracture types [19]. As demonstrated in this study by Zhang et al., among the 33 cases, 20 cases had lateral walls and lesser trochanter fragments at the same time, which accounted for 60 % of the total cases.

In addition,the terminology of basicervical trochanteric fracture is mentioned in this paper. Debates still hold relating to the unified nomination of it [20, 21]. However, the definition has been clearly explained as proximal femur fractures through the base of the femoral neck at its junction with the intertrochanteric region [22]. This relatively rare fracture type is characterized by its greater fracture angle, lack of muscular insertion and lack of cancellous interdigitation at the fracture line, and subsequent greater varus moment [20–23]. Consequently, it will become difficult to achieve posteromedial cortex continuity intraoperatively, i.e., calcar femorale integrity. Therefore, It has also been generally recognized that this type of fracture is inherently unstable.

### Possible mechanisms for wedge and reverse wedge effect

Contrary to the reverse wedge effect demonstrated in this study, Hu et al. found a “v” effect in basicervical intertrochanteric fracture after insertion of a nail’s massive empennage, which is quite similar to the abovementioned wedge effect [20]. Therefore, similar fracture types present different fragment migration effects, while different fracture types can have similar migration effects. This indicates that the nail insertion-related fragment migration effect may be closely related to intraoperative manipulation techniques, instead of specific fracture types, which need to be further explained.

**Fig. 1:**
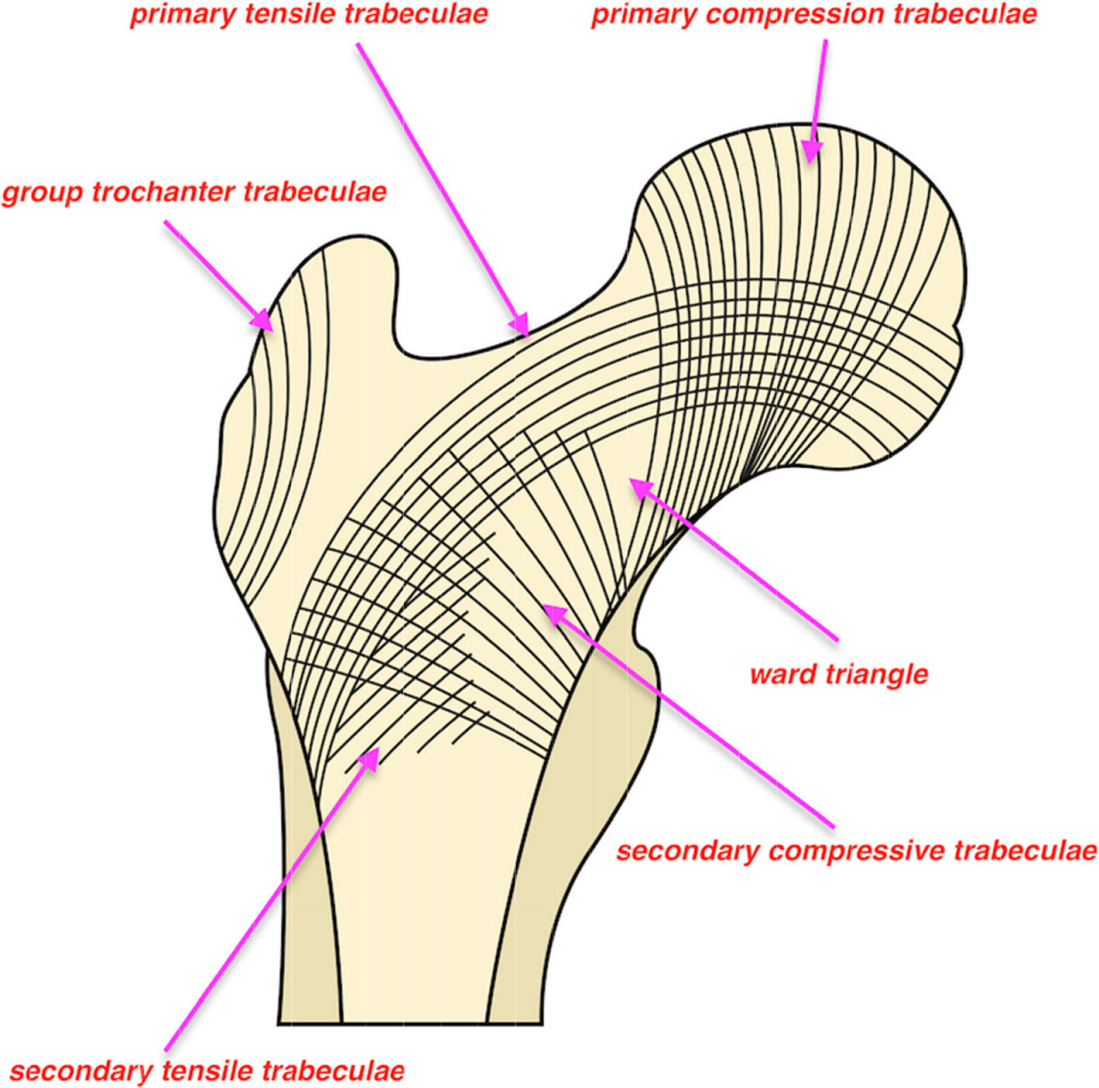
Schematic diagram defining normal trabeculae patter of proximal femur

**Fig. 2:**
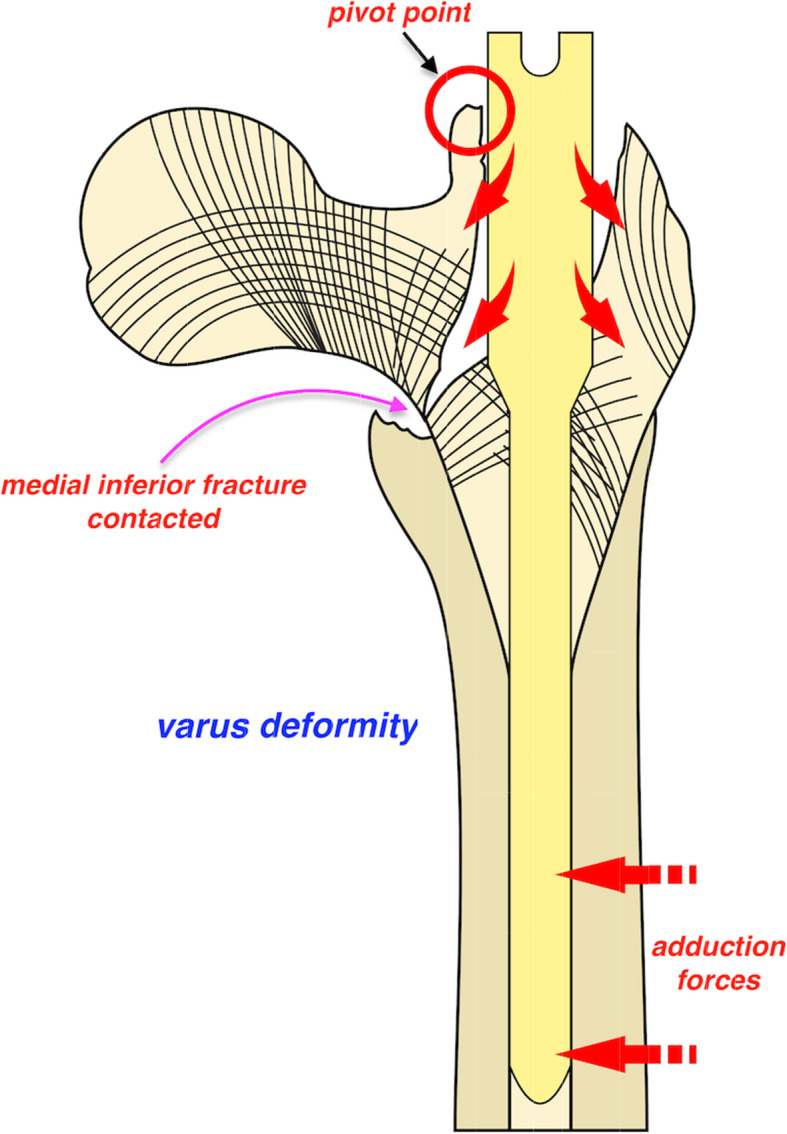
Schematic diagram defining underlying mechanism of wedge effect

**Fig. 3:**
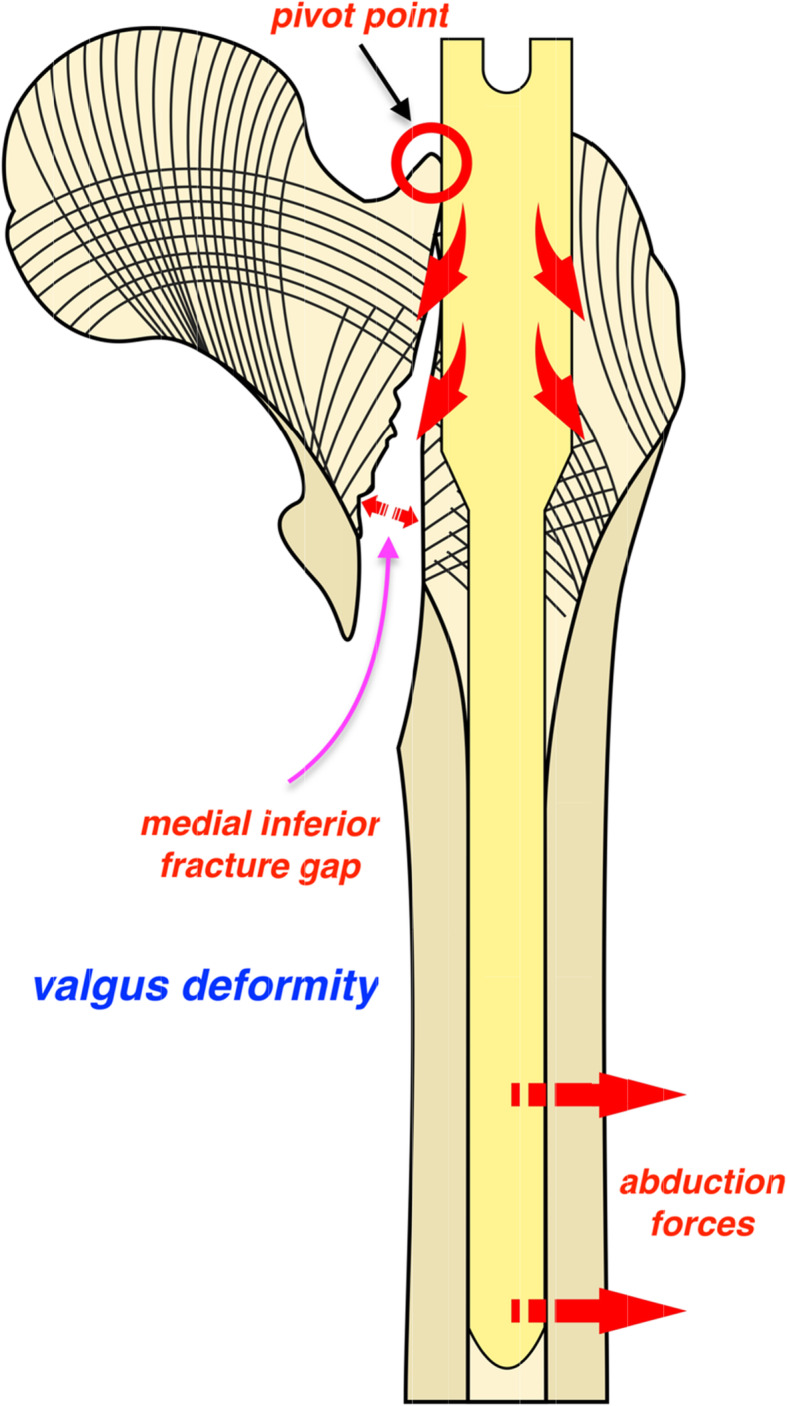
Schematic diagram defining underlying mechanism of reverse wedge effect

The bone structure at both sides of the proximal trochanter fracture site is different. The superlateral part of the femoral neck fragment is where the primary tensile trabeculae courses and will be disrupted after fracture occurrence [17]. The direction was horizontal and perpendicular to the longitudinal axis of the proximal femur (Fig. 1). Due to the weaker cancellous bone structure at the great trochanter, an outward pushing force would unavoidably be generated medially to laterally when the nail contacts the superolateral cortex of the femoral neck fragment during nail insertion into the medulla. If the great trochanter or lateral cortex is compromised, the location of the nail would be further lateralized accordingly. This should be the mechanism by which femoral shaft lateralization has been suggested to be a common phenomenon in various fracture types with wedge effects [5]. Different from varus reduction in the wedge effect, valgus reduction was observed in the reverse wedge effect in this study. As already proven, slight valgus reduction (< 10°) is favourable for interfragmentary reduction and better functional outcome. However, this could not be achieved without good contact of anteromedial cortices [7]. Nevertheless, a gap appeared at the medial inferior fracture line in the reverse wedge effect, which indicated that the anteromedial cortex apparently breached. The reason for this can be speculated as a principle of leverage acted by proximal femur on the pivot, i.e., contact point between the superolateral cortex of the femoral neck and the proximal end of the nail. Due to the proximally excessive space created during nail insertion, i.e., femoral shaft lateralization, the anteromedial cortex would either contact varus deformity, i.e., wedge effect, or breach in valgus, i.e., reverse wedge effect (Figs. 2 and 3). Unfortunately, this issue has not been explored in this cohort with a reverse wedge effect [8].

## Conclusions

Intraoperative assessment of reduction quality during trochanteric fracture operation is critical to achieve good fracture fixation and good functional outcomes. In this paper, up to 27 % of the total cases (12 cases in a total of 45 cases) did not show a reverse wedge effect intraoperatively. Therefore, whether this kind of reverse wedge effect is specific to the basicervical intertrochanteric fracture type needs to be analysed with more extensive studies. Regardless of the wedge effect, the reverse wedge effect or even the “V” effect will be met intraoperatively, and the key point is how to predict it preoperatively. Then, effective, convenient, and suitable intraoperative manoeuvres could be prepared during preoperative planning. Therefore, further efforts should be focused on the analysis of pre- and intraoperative related factors that could affect the intraoperative occurrence of wedge or reverse wedge effects and finding highly specific measures to overcome them.

## Data Availability

The datasets used and/or analysed during the current study are available from the corresponding author on reasonable request.

## Abbreviations

CT: Computed tomography
AO: Arbeitsgemeinschaftfür Osteosynthesefragen
OTA: Orthopaedic Trauma Association
IN: Intramedullary nail
DHS: Dynamic hip screw
ROC: Receiver operating characteristic
